# Environmental Exposures in Singapore Schools: An Ecological Study

**DOI:** 10.3390/ijerph18041843

**Published:** 2021-02-14

**Authors:** Divya Periyakoil, Hari Prasanna Das, Clayton Miller, Costas J. Spanos, Ndola Prata

**Affiliations:** 1 UC Berkeley Department of Electrical Engineering and Computer Science, Berkeley, CA 94720, USA; hpdas@berkeley.edu (H.P.D.); spanos@berkeley.edu (C.J.S.); 2 School of Design and Environment, National University of Singapore, Singapore 119077, Singapore; clayton@nus.edu.sg; 3 UC Berkeley School of Public Health, Berkeley, CA 94704, USA; ndola@berkeley.edu

**Keywords:** environmental, thermal comfort, children’s health, pollution, academic performance, school, population health, ecological study

## Abstract

Global climate change is a clear and present danger to our environment, but the impacts of climate change on human health are less known. People in Asian countries are more susceptible to the negative impacts of climate change and the subsequent environmental exposures because of the high population density, rapid urbanization, and natural geography of the region. The objective of this multidisciplinary collaborative ecological study was to explore the impact of environmental exposures such as temperature (°C), noise (db), humidity (%rh), air conditioning exposure time (hours), and distance traveled to school (km) on the comfort and academic success of school children in Singapore. Analysis of a large dataset from the Singapore National Science Experiment revealed a positive correlation between the distance traveled to school and favorable environmental conditions (moderate temperatures, low noise, low humidity, and higher amount of air conditioning time) and student academic performance. The analysis revealed that the distance traveled between home and school for public school students falls within a larger range than that for independent (private) school students. On average, students traveled farther distances to attend schools of higher academic caliber thereby increasing their exposure to environmental pollution. Student exposure to pollution can be minimized if all schools adhere to higher standards of environmental comfort and standardized academic caliber. If students can attend the school closest to their homes, they can minimize their daily pollution exposure due to traffic/commute, thereby mitigating the resultant negative health consequences.

## 1. Introduction

It is well known that global climate change is a clear and present danger to our environment and living conditions. However, less is known about the impacts of climate change and the subsequent environmental exposures on human health and disease. Experts feel that some existing health threats will intensify and new health threats will emerge [1]. Asian countries are particularly susceptible to the negative impacts of climate change. The United Nations Framework Convention of Climate Change stated that Asia is vulnerable because of the large and growing population, rapid urbanization, and the natural geography of the region [2]. Between 1901 and 2005, the surface temperatures of the regions of Asia and the Pacific Islands rose by 0.5–1.1 °C, and they have continued to rise since then, burdening the residents of Asian countries with a slew of negative effects [2]. The steady increase in heat is a public health threat, especially on vulnerable sectors of the population including children and older adults [2,3]. Exposure to excessive heat has been tied to risk of cardiovascular diseases, respiratory failure, asthma, and even premature mortality. Studies have also shown that the effects of heat and other environmental exposures can impact the education of students by affecting their cognitive performance and by hindering their learning processes through the exacerbation of respiratory health problems and by causing attention deficit and fatigue [4,5,6]. The Republic of Singapore, is a sovereign city-state in Southeast Asia. As an industrial country close to the Equator, it experiences pollution and exposure to seasonal smoke from Indonesian forest fires. Thus, in this island country with a 100% urban population, residents face the direct effects of pollution and heat [2].

The American Public Health Association (APHA) has been vocal about the global, negative impacts of environmental exposures that children face in school, stating that child behavior and physiology make them more susceptible to these exposures [7,8]. Children may not have the ability to identify pollution exposures or to communicate the presence of these exposures to an adult. The APHA has also stated that socioeconomic and cultural factors, in conjunction with the exposures, can play a role in creating disparities between children based on the quality of their environment [7,8]. The National Institutes of Health (NIH) has stressed that many pediatric health conditions are associated with pollution and heat and that these effects may be difficult to mitigate, especially in low-income countries [9]. Additionally, studies have shown that pollution exposure occurs not only in the school environment but also during the daily commute between home and school [5,10]. Indoor and outdoor air quality can have a direct effect on child respiratory health [5,10].

A recent study conducted on university students taking online courses amidst the COVID-19 pandemic demonstrated that noise, lighting, and temperature all have a profound negative effect on students’ academic outcomes as well as their comfort and concentration [11]. Another study showed that the school environment can have a strong effect on student academic performance and comfort and that ergonomic design of classrooms contributed to higher student performance and well-being [12]. Studies have also shown that the length of time traveled to school adds to the active natural and built environmental exposures on children and that environmental pollution exposure is impacted by the distance traveled from home to school [13,14]. It is important to examine the effects of environmental exposures on the health and education of school students, to inform future research concerning how to better support students in their school environment.

## 2. Objective

The objective of this ecological study was to examine the effects of various environmental exposures on Singapore schools and how these exposures varied with respect to gender, grade, whether the school is public or independent, and the average distance traveled from home to school. We analyzed a large volume of environmental data collected through a national study from school students in the Republic of Singapore with the goal of understanding the relationships between environmental comfort, environmental exposures in and out of school, and access to quality education.

## 3. Materials and Methods

### 3.1. Data Collection

#### 3.1.1. Environmental Exposure Data

The de-identified data used in this study were provided by the National Science Experiment (NSE) and used after securing appropriate permissions from the concerned agencies [15,16,17]. The National Science Experiment is an ongoing nationwide experiment (that began in 2015) conducted by the Singapore government. Singapore school students are provided a small wearable device that collects data that describes the state of their environment and their behaviors, such as information about temperature (C), noise (db), relative humidity (%rh), school and home locations, and steps traveled per hour. We analyzed the data from January-December 2016, which contained hourly environmental and behavioral data of 35,536 primary, secondary, and post-secondary school students in Singapore.

#### 3.1.2. School Data

We also utilized an NSE dataset containing the school attended by each of the students. By merging this dataset with the environmental dataset and aggregating the data by school, we were able to create a master dataset containing the average values of each of the environmental exposures (temperature, humidity, noise, and distance traveled between home and school) for each school. We also determined whether the schools were all-girls, all-boys, or co-educational institutions, as well as whether the schools were independent (private) or public schools. The final dataset consisted of 8 all-girls schools, 7 all-boys schools, and 104 co-educational schools. Of the schools, 24 were independent schools and 95 were public schools.

#### 3.1.3. School Rankings

The primary school education system of Singapore is structured in the following manner. Students are required by law to attend primary school for six years, approximately from ages six to twelve. At the end of primary school, students take the Primary School Leaving Examination (PSLE), and, until 2019, their scores would be a major deciding factor for the secondary school they would attend [18,19].

To infer the relative rankings of the secondary schools, we used the official PSLE cutoff scores for secondary school admissions. Although the concept of PSLE exam score cutoffs has since been removed as a criterion for secondary school admissions, we used PSLE data from before 2016 as our public health indicator, since the exam score cutoff rankings provided insight into the caliber of the school.

A limitation that we faced is that the PSLE cutoff established by any secondary school depends on the PSLE results of students who come from any of the numerous primary schools in Singapore. Although the PSLE score cutoffs for admission may be an indirect indication of the caliber of the school, the scores are not directly indicative. Rather, they are indicative of the caliber of the students who were admitted to the school, not the ones who are currently enrolled in the school. As a result, instead of using the 2016 score cutoffs, we averaged the score cutoffs for the years 2012, 2013, 2014, and 2015 [20,21,22,23].

### 3.2. Methods

Our study complies with research and publication ethical guidelines, as the purpose of the study was explained to all respondents to assure them anonymity and confidentiality. For data curation, cleaning, and analysis, we used the Python libraries Pandas, NumPy, and Scikit-Learn. Since we were conducting an ecological study, we aggregated the data by school, rather than examining individuals. For each school, we averaged the environmental exposures and distances traveled between home and school for every student attending that school. We calculated the average distance traveled by calculating the home-to-school distance for each of the students and averaging the results for every student that attended a particular school. We then added to the dataset relevant information regarding the gender of the schools and whether they were public or independent schools. We chose to examine four environmental exposures that are highly descriptive of the humid, hot, urban environment of Singapore: noise (db), temperature (C), humidity (% rh), and air conditioning exposure time (hours) [24,25,26]. We explored the relationships between (1) environmental exposures and distance traveled from home to school and (2) environmental exposures and academic caliber of the school. We did so by plotting each environmental exposure against distance traveled and academic caliber and performing linear regression. We examined these relationships across all schools as well as in smaller cohorts of gender and independent/public, to gain insight into how demographics can affect environmental and educational quality.

#### 3.2.1. Environmental Exposures and Distance Traveled

We wanted to understand the relationship between each of the four environmental exposures and the average distance traveled between home and school. We plotted the average environmental exposure values for all of the schools against the average distance traveled between home and that school. We subsequently plotted the environmental exposures against distance for smaller cohorts (with respect to gender and independent/public) of schools in order to understand how these relationships change with respect to demographics.

#### 3.2.2. Environmental Exposures and Academic Performance

We studied the relationships between environmental exposures and academic performance as quantified by the academic rank of the 35 secondary schools in the dataset. We used only secondary schools to do this analysis because secondary schools in Singapore are definitively ranked, and those rankings are available publicly. We used the PSLE score rankings to order the secondary schools by rank and then plotted the environmental exposure levels and distance traveled from home to school against the rank.

## 4. Results

### 4.1. Environmental Exposures and Distance Traveled

#### 4.1.1. Aggregate School Data

**Figure 1:**
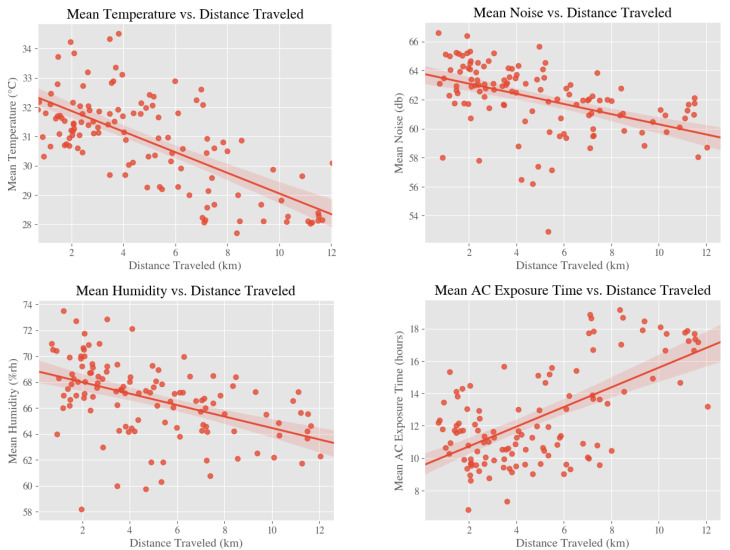
Relationships between environmental exposures and distance traveled between home and school.

As shown in Figure 1, we found that mean distance traveled between home and school was inversely correlated with mean noise exposure (r = −0.469, *p* < 0.001), mean temperature (r = −0.682, *p* < 0.001), and mean humidity (r = −0.467, *p* < 0.001) and directly correlated with mean AC exposure time (r = 0.624, *p* < 0.001).

#### 4.1.2. Gender

**Figure 2:**
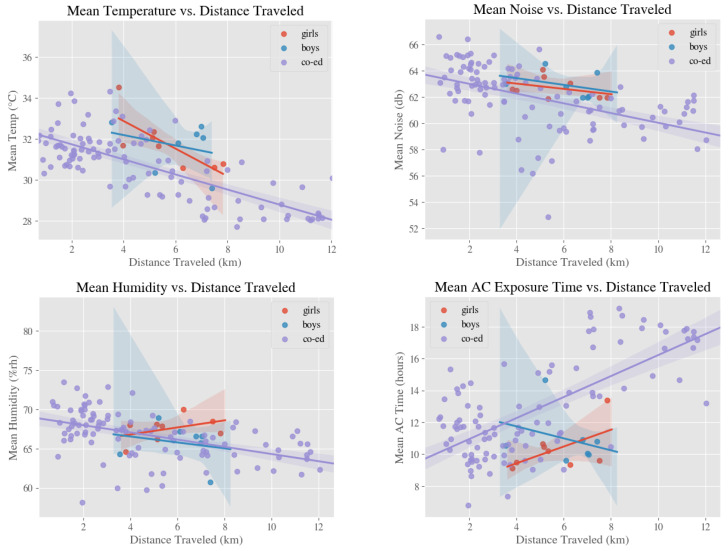
Relationships between environmental exposures and distance traveled between home and school with respect to the gender of the school student body.

Figure 2 and Table 1 show the relationships between distance traveled from home and school and various environmental exposures for all-girls (eight schools), all-boys (seven schools), and co-ed schools (104 schools). Although mean temperature and mean noise were inversely correlated with distance traveled from home to school for all three categories, there were some discrepancies among the categories with respect to mean humidity and mean AC exposure time. These results could be attributed to the fact that the dataset was imbalanced with respect to gender. The dataset had eight all-girls and seven all-boys schools, but 104 co-ed schools. From Figure 2, the most striking difference between single-gender schools and co-ed schools was that the range of distance traveled to school for co-ed schools was much wider than that of single-gender schools. This difference can be attributed to the fact that seven out of the eight all-girls schools and all all-boys schools in the dataset were independent schools.

#### 4.1.3. Independent/Public

**Figure 3:**
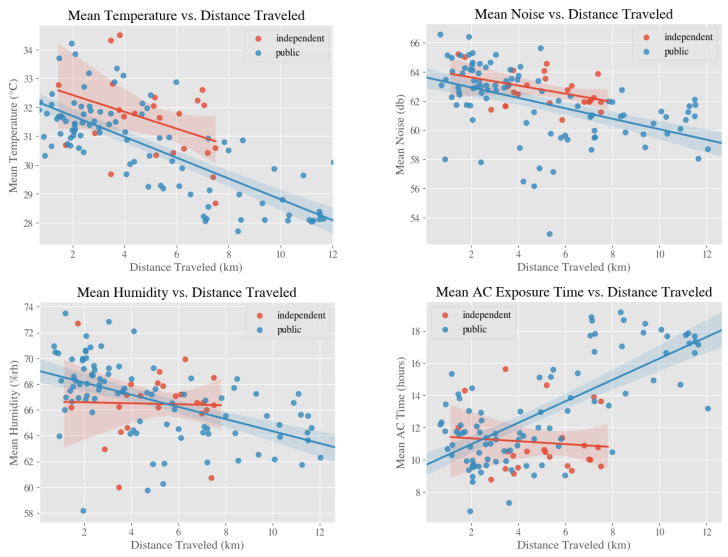
Relationships between environmental exposures and distance traveled between home and school with respect to whether the school is a independent or public school.

We found that for both independent (*n* = 24) and public schools (*n* = 95), the distance traveled from home to school by students was inversely correlated with mean temperature (independent: r = −0.381, *p* = 0.066; public: r = −0.753, *p* < 0.001) and mean noise (independent: r = −0.418, *p* = 0.042; public: r = −0.483, *p* < 0.001), as shown in Figure 3. In public schools, the distance traveled to school was inversely correlated with mean humidity (r = −0.527, *p* < 0.001) and directly correlated with mean AC exposure time (r = 0.714, *p* < 0.001). In independent schools, there was no discernible association between distance traveled to school and mean humidity (r = −0.027, *p* = 0.901) or distance traveled to school and mean AC exposure time (r = −0.085, *p* = 0.691). As shown in Figure 3, the range of distance from home to school traveled by public school students was also far wider than that traveled by independent school students, implying that public school students may have to travel farther to school.

### 4.2. Environmental Exposures and Academic Performance

**Figure 4:**
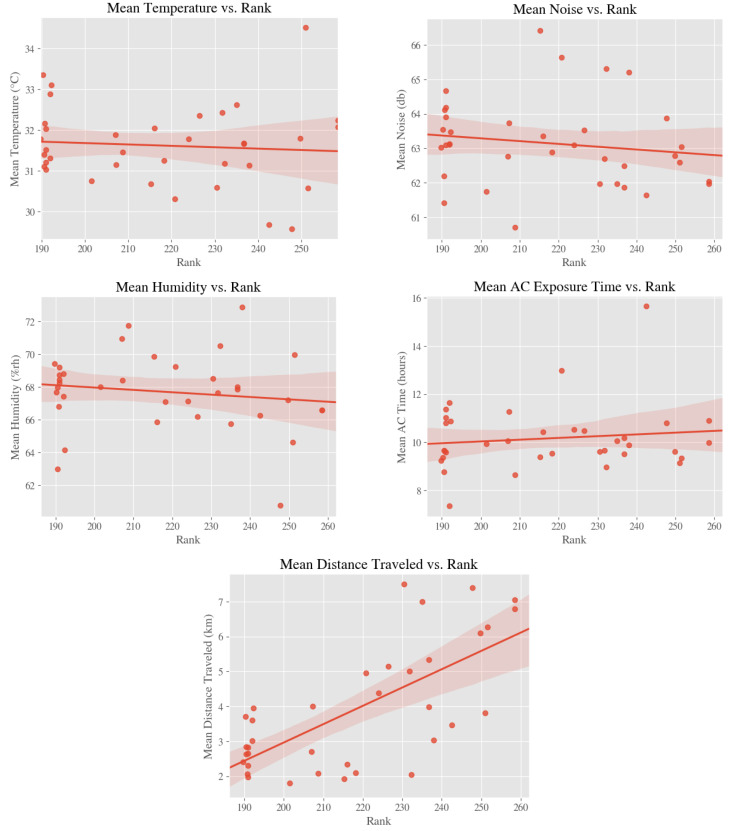
Relationships between environmental exposures/distance and school academic rank.

As shown in Figure 4, we found that rank of the 35 secondary schools tended toward inverse correlation with mean temperature (r = −0.081, *p* = 0.637), mean noise (r = −0.15, *p* = 0.37), and mean humidity (r = −0.145, *p* = 0.398). Rank also tended toward direct correlation with mean AC exposure time (r = 0.125, *p* = 0.467) and mean distance traveled (r = 0.694, *p* < 0.001). From these results, we observed a strong correlation between the distance traveled between home and school and the rank of the school, implying a perception of higher academic quality in schools at a farther distance from home.

## 5. Discussion

This work is unique because it is a large study involving 35,536 students studying in 119 schools in the Republic of Singapore. Of these schools, eight schools are all girls, seven schools are all boys, and 104 schools are co-educational schools. Additionally, of these schools, 24 are independent and 95 are public schools. We plotted each of the exposures against both academic performance and distance traveled to investigate the relationships between environmental comfort (as quantified by temperature (°C), noise (db), humidity (%rh), and air conditioning exposure time (hours) and academic success (based on PSLE scores)) in Singaporean school children from diverse backgrounds. This study represents the first time that NSE data has been used to better understand primary, secondary, and post-secondary school student health.

Our data showed that overall, school students were likely to travel greater distances to attend schools of high environmental comfort and strong academic caliber. We also found that compared to public school students, students who attended independent schools typically traveled shorter distances between home and school. While public school students traveled anywhere between 2 and 12 km between home and school, independent school students traveled between 2 and 8 km between home and school. These findings imply that students from lower socioeconomic status likely attended public schools and had to travel farther to attend a school of higher quality. As demonstrated in previous studies, as children have to travel farther to attend school, they will be exposed to more pollutants and will become more tired compared to those who attend school closer to their homes [10,27]. Studies have shown that traffic-related air pollution can have dire consequences on student health and that schools must not only choose areas of low pollution but must also consider the effects of pollution exposure through transportation and traffic [10,27].

To combat inequity in access to education, we need to implement a system by which a high quality of education is available through every single school in Singapore. This system will ensure that children can go to local schools close to their homes. Fortunately, the government of Singapore has already taken a strong step toward equity in education by removing emphasis from the PSLE exam scores in order to place more emphasis on student well-being. As PSLE exam scores no longer inform the caliber of school that a student will attend, it can be assumed that the standard of education in schools will normalize and become more equivalent to one another. Quality of education and quality of a student’s thermal environment should not be contingent on the distance traveled to reach school [10,27,28].

Finally, environmental comfort should be held to the highest standard. Due to the urban air pollution and noise pollution present in Singapore, schools must institute the highest quality of environmental comfort, to ensure a conducive learning environment for students. As suggested by a 2020 study in Mexico about how environmental exposures (light, noise, and temperature) affect student performance, ergonomically designing classrooms to provide optimal temperature, humidity, and noise levels would be a strong step towards improving environmental comfort standards in schools [11].

The imbalanced nature of the dataset with respect to gender was a limitation of this study and made it difficult to reach definitive conclusions concerning the relationship between gender and equity in access to quality education. In the future, we hope to further explore how gender and socioeconomic factors play a role in gaining access to quality education. We also hope to continue this work and learn how distance traveled between home and school and the related environmental exposures may be affecting student health and school absences.

## 6. Conclusions

According to the World Health Organization (WHO), between 2030 and 2050, the direct and indirect effects of climate change and the subsequent environmental exposures are expected to cause approximately 250,000 additional deaths per year. Singapore’s location and urbanization make it particularly susceptible to these negative impacts [2]. Child behavior and physiology put students at additional risk, as do socioeconomic factors [1]. The negative impacts on children of traffic pollution exposure from the home to school commute are disproportionately high, especially on those children from lower socioeconomic strata [1]. Environmental exposures in schools can also have lasting detrimental effects on the health of children, such as on brain development and respiratory health [7,8]. Thus, it is essential to work toward providing equitable access to a high quality of environmental comfort for students in the school environment, and normalizing education standards in schools across Singapore. These measures will ensure that students can attend the school closest to them without worrying about the quality of the education that they are receiving [1]. The first step towards doing so is understanding the relationships between environmental comfort and academic success. Through this multidisciplinary, collaborative ecological study, our work has created a framework for understanding how the school environment relates to the comfort and academic success of school children, which we hope will inform future research. 

## Figures and Tables

**Table 1:** Correlation between environmental exposures and distance to school with respect to gender.

(a) Mean Temp vs. Distance Traveled		
	r	*p*
boys	−0.286	0.534
girls	−0.771	0.025
co-ed	−0.741	<0.001
(b) Mean Noise vs. Distance Traveled		
	r	*p*
boys	−0.342	0.453
girls	−0.369	0.369
co-ed	−0.494	<0.001
(c) Mean Humidity vs. Distance Traveled		
	r	*p*
boys	−0.197	0.672
girls	0.413	0.309
co-ed	−0.493	<0.001
(d) Mean AC Exposure vs. Distance Traveled		
	r	*p*
boys	−0.300	0.514
girls	0.567	0.142
co-ed	0.694	<0.001

## Data Availability

Due to the nature of this research, the Government of the Republic of Singapore has not given consent for the data to be shared publicly, so supporting data is not available.
